# A Diary-Based Study of Language Use During Cognitive Decline

**DOI:** 10.1093/geroni/igaa057.1910

**Published:** 2020-12-16

**Authors:** Katharina Pabst, Sali Tagliamonte

**Affiliations:** University of Toronto, Toronto, Ontario, Canada

## Abstract

Although the effects of dementia on language have been examined in a variety of ways, there is relatively little data available showing longitudinal effects. In the present study, we examined the effect of cognitive decline on an individual’s language use over time (from age 60-91) by assessing daily diary entries. We focused on a linguistic phenomenon commonly associated with diaries and other informal registers, i.e. subject omission, as in “∅ Made cranberry muffins.” Quantitative analysis revealed a drastic decline in the use of this feature, which is correlated with the stages of the individual’s cognitive decline. We argue that the patterns reflect a systematic reduction in complexity that affects a writing style based on literacy and acquired at a later stage than more basic writing. This corroborates previous findings, showing later-acquired linguistic features are disproportionately affected in cognitive decline and highlights the importance of linguistic analysis in aging research.

